# Deferiprone: A Forty-Year-Old Multi-Targeting Drug with Possible Activity against COVID-19 and Diseases of Similar Symptomatology

**DOI:** 10.3390/ijms23126735

**Published:** 2022-06-16

**Authors:** George J. Kontoghiorghes

**Affiliations:** Postgraduate Research Institute of Science, Technology, Environment and Medicine, Limassol 3021, Cyprus; kontoghiorghes.g.j@pri.ac.cy; Tel./Fax: +357-26272076

**Keywords:** COVID-19, SARS-CoV-2, deferiprone, drug design, drug targeting, multitarget drugs, health strategies, drug combinations

## Abstract

The need for preparing new strategies for the design of emergency drug therapies against COVID-19 and similar diseases in the future is rather urgent, considering the high rate of morbidity and especially mortality associated with COVID-19, which so far has exceeded 18 million lives. Such strategies could be conceived by targeting the causes and also the serious toxic side effects of the diseases, as well as associated biochemical and physiological pathways. Deferiprone (L1) is an EMA- and FDA-approved drug used worldwide for the treatment of iron overload and also other conditions where there are no effective treatments. The multi-potent effects and high safety record of L1 in iron loaded and non-iron loaded categories of patients suggests that L1 could be developed as a “magic bullet” drug against COVID-19 and diseases of similar symptomatology. The mode of action of L1 includes antiviral, antimicrobial, antioxidant, anti-hypoxic and anti-ferroptotic effects, iron buffering interactions with transferrin, iron mobilizing effects from ferritin, macrophages and other cells involved in the immune response and hyperinflammation, as well as many other therapeutic interventions. Similarly, several pharmacological and other characteristics of L1, including extensive tissue distribution and low cost of production, increase the prospect of worldwide availability, as well as many other therapeutic approach strategies involving drug combinations, adjuvant therapies and disease prevention.

## 1. Introduction

A report by the World Health Organization (WHO) estimated that as of the end of February 2022, there have been globally about 0.5 billion confirmed cases of the coronavirus disease 2019 (COVID-19), including about 6 million deaths (according to another report, 18 million deaths) since the beginning of the pandemic [1,2]. It has also been estimated that more than 10 billion vaccine doses against the severe acute respiratory syndrome coronavirus 2 (SARS-CoV-2) have been administered over the same period [1].

Different health strategies have been adopted in each country for controlling and reducing the morbidity and mortality associated with COVID-19, based on different priorities and aims at each stage of the progression of the pandemic, including transport restrictions, financial growth, educational and public health considerations for the treatment of other diseases, etc. [3].

The general strategy for curbing the COVID-19 pandemic is based on the prevention of transmission of SARS-CoV-2 and the identification and development of drug(s) and vaccines, which can decrease the mortality rate to minimum acceptable levels [3,4,5]. In this context, measures preventing transmission of SARS-CoV-2—such as self-isolation, distancing between individuals and face masks, as well as vaccinations and the administration of different drugs for supporting the prospects of survival in selected categories of seriously ill patients—have so far been the mainstay therapy for the limited control of the spread of the infection and therapeutic prospects for decreasing the rate of mortality [3,5,6,7,8,9].

However, despite the periodical improvements and therapeutic successes, COVID-19 is still here and is life threatening for the whole of humanity because of many unsettling factors such as the slow development and low availability of effective antiviral drugs and vaccines, limited effect of vaccinations, increased prospects of reinfection after vaccination, more emerging toxic SARS-CoV-2 variants and insufficient prophylaxis from viral transmission and toxicity [3,5,10,11,12,13]. 

There are many emerging challenges for the development of drugs targeting different aspects of COVID-19. These include the targeting of different stages of the disease, such as transmission and proliferation, ‘hyper-inflammation/cytokine storm’, lung damage and multi-organ damage and sepsis, long-term side effects and many others [3,5,8,9,14,15]. Drug targeting could mainly be focused on the respiratory system, which is the most affected and major cause of mortality by COVID-19, but also other systems which are affected at a different extent such as the cardiovascular, gastrointestinal, nervous, immune and hematopoietic systems [3,5,7,14,15]. Furthermore, drug targeting could be based on different categories of patients which are more susceptible to SARS-CoV-2 infection such as obese, renal, pulmonary, diabetic, immune-compromised and cardiovascular patients [3,7].

The development of specific drugs with effective targeting in each of the above categories could increase the prospects of a better outcome in the COVID-19 pandemic. In this context, there can be different strategies and approaches in the selection and evaluation process for new drugs against the disease [3,5,15]. One such strategy could be based on the identification of one drug for one target, another based on a multi-potent drug for many targets and also drug combinations for one or more targets [3,5]. The aim of any strategy is the selection of one drug or drugs for achieving a significant reduction in mortality in COVID-19 to acceptable levels; e.g., similar to the mortality rate caused by the influenza virus [3,4,5,16].

Several drugs are regularly used in different categories of COVID-19 patients, especially those suffering from pulmonary complications and hypoxia. For example, one such drug is remdesivir, which has broad spectrum antiviral activity and was initially used against the hepatitis C virus. Remdesivir is a pro-drug, which is metabolized to a ribonucleotide analogue inhibitor of viral RNA polymerase. In clinical studies remdesivir has been shown to cause a reduction in the rate of mortality of hypoxic COVID-19 patients [17,18,19,20]. Two other drugs widely used for treating COVID-19 patients are molnupiravir and paxlovid. Molnupiravir was initially developed to treat influenza and also recently licensed to prevent severe COVID-19 infection in patients [21,22]. It is a pro-drug of a synthetic nucleoside and exerts its antiviral action through introduction of copying errors during viral RNA replication. Oral molnupiravir appears to reduce the risk of hospitalization and death from COVID-19 by about 50% for newly diagnosed, high-risk patients [22]. Another antiviral co-packaged medication is a drug combination of nirmatrelvir and ritonavir, sold under the brand name paxlovid. It is used for the treatment of mild-to-moderate COVID-19 patients, who are at high risk for progression to severe COVID-19, including hospitalization or death. Oral paxlovid has been shown to reduce hospital admissions and deaths by 80–90% among patients with COVID-19 who are at high risk of severe illness [23]. The mode of action of nirmatrelvir is inhibition of the activity of the SARS-CoV-2-3CL protease, an enzyme that the coronavirus needs to replicate. Co-administration with ritonavir decreases the metabolism of nirmatrelvir and maintains its antiviral activity at higher levels [23]. Dexamethasone and other corticosteroids are also widely used in certain categories of patients for inhibiting the immune system, including cytokine response during hypoxia [24]. Progress in COVID-19 research has been very rapid, including development of antiviral drugs which are effective at the early phase, and also immune-modulating agents for treating cytokine storm [3,5,17,18,19,20,21,22,23].

In the meantime, there are thousands of drugs and nutraceuticals along with their combinations that can be used to select candidate therapeutics for targeting the transmission, proliferation and the fatal or severe symptoms of SARS-CoV-2 [3,5]. The development of more such drugs could reduce further the unacceptably high morbidity and mortality rate observed in the COVID-19 pandemic, as well as its associated newly identified long-term side effects and overall negative effects on daily life worldwide [3,24,25,26,27,28,29,30,31].

**Figure 1:**
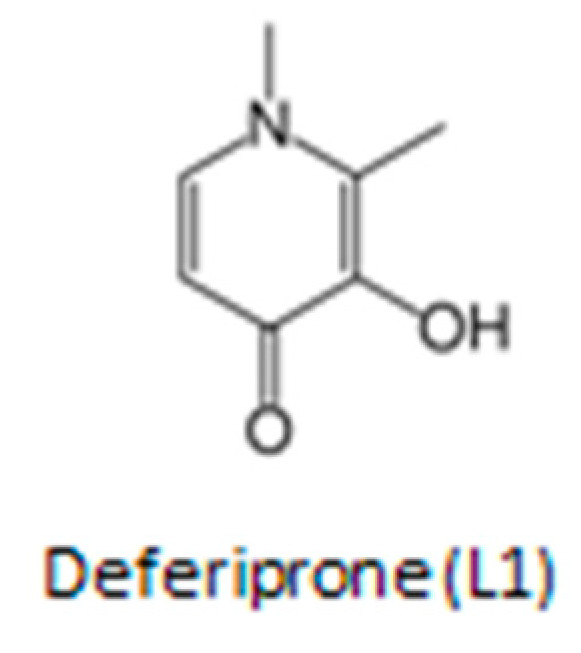
The chemical structure of the iron chelating drug deferiprone (L1). The drug is used mainly for the treatment of iron overload in thalassemia but also in many other diseases.

One such promising candidate drug against COVID-19 is deferiprone (L1), an iron chelating drug approved by the drug regulatory authorities, the FDA in the USA and EMA in the European Union, for the treatment of iron overload in thalassemia but has also been tested and shown to be effective in many other clinical conditions including viral infections (Figure 1) [3,32,33]. The drug was designed more than 40 years ago and tested in many in vitro, in vivo and clinical systems [3,32,33]. A detailed analysis of the clinical, pharmacological and other properties of L1 suggests that there may be an increased prospect for its use as a multi-potent drug against COVID-19 and also diseases with similar symptomatology. 

## 2. Pharmacological and Toxicological Considerations in the Use of Deferiprone against COVID-19

Drug selection against COVID-19 by the regulatory authorities is similar to that in many orphan drug development efforts, and in particular drug approval for emergency use in diseases where there are no available therapeutics [34]. Furthermore, the search for emergency drugs for the COVID-19 pandemic is considered urgent because of the rapid progress of the disease in different world regions and also the large number of the fatalities observed in aged and other susceptible categories of patients [3,5,6,7,8,35,36,37]. The prospect of the development of effective drugs for each of the different stages of COVID-19 is also challenging, since any drug targeting effectively one of the different targets or stages of the disease could potentially reduce the rate of mortality. Similarly, multi-targeting drugs such as L1 or drug combinations may further increase the prospects of mortality reduction in COVID-19.

Deferiprone is an EMA- and FDA-approved drug, which is listed by the WHO as one of the essential medicines (Figure 1). It has been used for more than 25 years by hundreds of thousands of patients worldwide for the treatment of transfusional iron overload in thalassemia and other conditions [32,33,34,38]. The general molecular characteristics, mechanisms of chelation, antioxidant, pharmacological, toxicological and other properties of L1 have been previously reviewed [32,33,34,38,39]. Deferiprone is an orally active, low molecular weight hydrophilic drug, which is on the top of the list of drugs with the highest safety record per dose and frequency of administration. It has been used in many non-iron loaded categories of patients in addition to iron overload categories with no significant toxicity [32,33,34,38,39]. Deferiprone can diffuse through almost all major organs where it can exert effectively its antiviral, antioxidant and other therapeutic effects [33,34,38,39].

The relatively short life cycle of SARS-CoV-2 and its associated life-threatening toxicity implications suggest that proposed therapeutic drugs against COVID-19 should exert their therapeutic activity in a matter of a few days or weeks [3,5]. The risk/benefit assessment for this short therapeutic time window can generally allow the administration of repeated high doses of L1 at the maximum dose of the approved range (50–100 mg/kg/day), similar to that in other non-iron loaded categories of patients. This short-term treatment period and the prior use of L1 in other categories of patients could potentially facilitate the rapid approval of drug trials and clinical use due to the emergency COVID-19 pandemic conditions, or other similar disease situations in the future [3,5,15,34].

### 2.1. Pharmacological Properties and Effects of Deferiprone

The pharmacological properties and other molecular characteristics of L1 have been under investigation in the past 40 years. Deferiprone is a small molecule of neutral charge, which is orally absorbed, widely distributed in most tissues and organs such as the heart, liver, spleen and the brain and is excreted almost completely in the urine [32,38,39]. It can also be monitored and measured in blood and also in saliva. Deferiprone can exert its therapeutic effects in most tissues, organs and cells as a result of its extensive tissue distribution. For example, L1 can enter most iron loaded tissues and mobilize excess iron by forming an iron complex of stoichiometry of three molecules of L1 to one molecule of iron. The iron complex of L1 has a red color similar to that excreted in the urines of iron loaded patients [32]. Although L1 has high affinity for iron, it can also bind other endogenous and xenobiotic metals including copper, zinc, aluminum, indium, plutonium, europium and uranium [40,41,42,43,44].

The pharmacokinetic and metabolic properties of L1 have been previously reported and some are shown in Table 1 [45,46,47,48]. Oral L1 is readily absorbed from the stomach, metabolized to a glucuronide conjugate in the liver, cleared from the plasma over a period of 6–8 h and excreted in the urine in three forms, namely the L1 iron complex, L1 glucuronide conjugate and free unconjugated L1 [38,45,46,47,48].

The interactions of L1 with iron and other metal ions on the molecular level, proteins of iron metabolism, cells and tissues has also been extensively studied in vitro and in vivo. Iron chelation and mobilization by L1 has been shown to occur from all the iron pools in cells including intracellular low molecular weight iron, ferritin and hemosiderin and also from transferrin and non-transferrin bound iron (NTBI) in plasma. It has also been shown in clinical studies that the mobilization of iron by L1 depends in general on the iron load of the patients and the dose of L1 [38,46]. The increase of urinary iron excretion caused by L1 in non-iron loaded categories of patients is only a few mg, which is a small fraction in comparison to the amount of iron excreted in iron loaded patients [46]. The level of iron that could be excreted during L1 therapy in non-iron loaded patients, e.g., COVID-19 patients, is negligible and less than the amount of iron present in western diets or less than 1% of what is lost during blood donation. In this context it is expected that the amount of iron removed in COVID-19 patients during the short period of treatment, e.g., a week, can easily be replaced by dietary iron.

The body distribution of iron and mode of iron removal activity by L1 and other chelating drugs from iron loaded and non-iron loaded categories of patients, as well as the determination of the iron metabolic pathways involved in these processes, can be determined using different diagnostic techniques [49]. In particular, the recent introduction of the magnetic resonance imaging (MRI) T2 and T2* techniques have been instrumental for identifying the level and distribution of iron load in the heart, liver, spleen, brain and other organs [50,51,52,53,54]. Similarly, the same MRI methods have been used for monitoring the efficacy, specificity and safety levels of iron removal from different organs by L1 and other chelating drugs [55,56]. This information is vital for toxicity monitoring and also for designing therapeutic strategies for specific targets, as well as for personalized therapeutic protocols in iron overload diseases and also other diseases of focal iron deposits [52,56]. 

The high efficacy and low toxicity of L1 in the treatment of iron overload prompted investigations of its use and development in many other clinical conditions, especially in conditions with no other effective therapies such as neurodegenerative, cardiovascular, renal, infectious diseases, cancer, as well as all diseases associated with free radical pathology including ageing and also currently COVID-19 [3,5,34,57,58]. 

In each case, the strategic initiatives for the use of L1 in non-iron loaded diseases were based on the risk/benefit assessment of therapeutic outcomes in each of these diseases, where no other effective therapies are available. The same approach is also adopted for the proposed multi-target treatment of COVID-19 using L1.

### 2.2. Toxicological Aspects of Deferiprone Therapy

The safety of L1 has been studied over the past 40 years in different in vitro, in vivo and clinical models of short- and long-term toxicity [33,59,60]. Most importantly, the safety of L1 has been confirmed in different categories of patients following short- and long-term studies, as well as continuous clinical monitoring involving thousands of iron loaded patients and also other groups of non-iron loaded patients in the past 35 years [33,57,58,59,60]. The most serious toxic side effects observed during short- and long-term treatment in iron loaded thalassemia patients are agranulocytosis (1% >) and neutropenia (5% >). Both toxicities are reversible and weekly or fortnightly mandatory blood count monitoring is recommended for prophylaxis for all patients using L1. Less serious toxic side effects include musculoskeletal and joint pains, gastric intolerance and zinc deficiency [33,59,60].

Prophylactic measures and toxicity vigilance are usually necessary and implemented for monitoring the safety of L1 and also of other drugs during long-term treatments. In this context, mandatory monitoring of weekly white blood cell count is considered as an essential prophylactic measure for the prevention of agranulocytosis during long-term treatment with L1. In cases of L1 agranulocytosis, the toxicity is transient and recovery is achieved following treatment with granulocyte-colony stimulating factor (G-CSF) [33,61]. The mechanism of L1-induced agranulocytosis is thought to be related to an immune response against white cell progenitors produced in the bone marrow. Zinc supplementation is also used for prophylaxis in patients on long-term treatment with L1 [33]. It can be considered that in general, all the toxic side effects of L1 during long-term clinical use are considered controllable, manageable and reversible. 

The serious toxic side effects reported during L1 treatments in chronic cases are not expected to appear during short-term treatments, such as those using other drugs in COVID-19 patients. The absence of toxic side effects has also been previously shown during L1 short treatment periods in other categories of non-iron loaded patients.

No serious toxic side effects have in general been observed regarding the posology of L1 in iron loaded and non-iron loaded patients. The range of doses for the proposed use of L1 in COVID-19 patients depends on several parameters including the target characteristics of different aspects of the disease. For example, low doses (10 mg/kg/day) could be used for prophylaxis, whereas maximum doses (100 mg/kg/day) could be used for intensive therapy protocols [62,63]. In considering tolerance, divided doses of L1 of up to 250 mg/kg/day in total have been used in intensive chelation in iron loaded thalassemia patients causing continuous increases in iron excretion and with no apparent toxicity [33,46].

Another parameter of possible toxicity in the use of L1 in COVID-19 patients is the monitoring of serum ferritin levels. Despite that serum ferritin increases are observed in seriously ill COVID-19 patients during the inflammatory response, the changes are not a reflection of an increase in the iron stores. In contrast, decreases of serum ferritin levels in thalassemia major patients reflect a lowering of the iron store levels, and in some cases of low serum levels withdrawal of chelation therapy may be necessary for avoiding iron deficiency [64,65]. However, no substantial decreases of serum ferritin or body iron load can possibly occur during a course of chelation therapy of a short period, e.g., a week in COVID-19 patients.

Overall it can be suggested that there is a very low prospect of toxicity, including insignificant reduction of the iron stores during a short course, e.g., a week’s treatment for COVID-19 patients treated with L1.

## 3. Deferiprone Targeting Molecules and Metabolic Pathways of COVID-19

There are many clinical and other characteristics, in addition to the pharmacological and safety features of L1, which may play a role in the targeting process and therapeutic prospects against COVID-19 and related diseases. The targeting features have been identified from several findings during the clinical use of L1 and other iron chelating drugs in clinical conditions of similar symptomatology to COVID-19, where there was no available effective drug treatment and also a high morbidity and mortality rate.

The introduction of L1 for the treatment of many iron loaded and also non-iron loaded categories of patients had in each case different characteristics and pathophysiological effects, which in most but not all such cases were related to iron metabolic pathways and associated toxicity or functional activity abnormalities.

### 3.1. Iron Chelation and Antioxidant Effects of Deferiprone in Relation to COVID-19

Iron toxicity, which originates from different forms of excess iron observed in vivo, including iron overload, low molecular weight iron forms and some protein bound iron forms, is considered as a potential hazard and a negative prognostic factor for all diseases [66]. A major target of antioxidant therapies includes the control of iron, which is the most common biological catalyst of free radical reactions and is associated with almost all diseases of free radical pathology [67,68,69,70]. Within this context, the iron chelation-antioxidant effects of L1 were first identified and developed in 1987 and its potential application in a wide range of associated iron loaded and non-iron diseases, including viral diseases, was proposed in 2003 [68,71].

One of the major characteristics and toxic side effects of COVID-19 is the extensive endothelial and other tissue damage which affects mainly the lungs and the heart, but also other organs in susceptible patients during hyper-inflammation or the “inflammatory storm” [15,72]. Within this context, a vicious cycle of oxidative stress toxicity including ferroptosis, which mainly involves free radical damage catalyzed mostly by iron, is considered as one of the major factors involved in the tissue damage following the inflammatory storm and during hypoxia, leading to fatalities in COVID-19 [3,15]. The same form of oxidative stress toxicity leading to tissue damage has been proposed in many other clinical conditions including neurodegenerative diseases, ischemia/reperfusion damage, diabetic and non-diabetic glomerular disease and many iron overloading conditions [67,70,73,74,75,76]. Different antioxidant treatments have been proposed in many clinical conditions related to free radical pathology in the past 50 years, including iron chelation-antioxidant therapy where in vitro and in vivo studies have shown encouraging results. Based on these preclinical antioxidant effects, L1 was selected for clinical studies in different categories of patients as a reflection of its iron chelation-antioxidant and safety potential.

In extensive clinical investigations and monitoring of iron loaded thalassemia patients treated with L1, it was observed that removal of excess toxic iron from the heart resulted in substantial improvement of cardiac function. In particular, it was observed that the long-term use of L1 significantly enhances left-ventricular ejection fraction (LVEF) and improves the oxidative status in thalassemia major patients [77,78]. Similar effects were observed in other categories of patients with cardiac problems caused by iron overload [79,80]. On the cellular level, the improved left-ventricular ejection function was suggested to be related to the iron chelation−antioxidant effects of L1 on endothelial cells [81]. Improvements in oxidative status and function including increases in glutathione levels were also observed in the red blood cells of iron loaded patients treated with L1 [82].

The therapeutic effects of L1 are also evident in many categories of patients with different underlying pathologies but with normal iron store levels, including diagnostic parameters such as serum ferritin. For example, serious toxicity has been observed in many non-iron loaded categories of patients, where the presence of focal iron deposits can be detected by MRI T2*. The displacement of iron and accumulation of iron deposits in tissues leading to damaging effects has been implicated in many diseases of free radical pathology. In particular, iron accumulation in the brain with increased signal intensity of MRI T2* has been detected in many neurodegenerative diseases including Friedreich’s ataxia, Parkinson’s disease, Alzheimer’s disease and neurodegeneration with brain iron accumulation (NBIA) [83,84,85,86].

Neurodegeneration with brain iron accumulation is a heterogeneous group of progressive neurodegenerative diseases characterized by iron deposition in the globus pallidus and the substantia nigra of the brain. At least 15 diseases have been identified in this group, including the four most common forms, which are pantothenate kinase-associated neurodegeneration (PKAN), phospholipase A2 group VI (PLA2G6)-associated neurodegeneration (PLAN), beta-propeller protein-associated neurodegeneration (BPAN) and mitochondrial membrane protein-associated neurodegeneration (MPAN) [87]. Similarly, toxic labile iron has also been implicated in many other diseases of free radical pathology in addition to neurodegenerative diseases, e.g., in diabetic and non-diabetic glomerular disease, ischemia reperfusion injury, etc. (Table 2) [67,70,73,74,75,76]. Many clinical studies have been carried out for the selection and administration of appropriate doses of L1, which in most cases have resulted in significant clinical improvements in all these different categories of patients.

The iron chelation–antioxidant effects of L1 in so many different clinical conditions are a major indicator regarding the safety and potential prospects for use in other conditions with increased rate of tissue damage including COVID-19. The antioxidant activity of L1 in COVID-19 for the minimization of tissue damage in the lungs, the heart and other organs could be enhanced by antioxidant drug combinations targeting different molecules or pathways of oxidative stress toxicity, such as combination of L1 with N-acetylcysteine [58].

Clinical trials for the evaluation of the therapeutic effects of L1 in non-iron loaded patients with focal iron deposits, including Parkinson’s and Alzheimer’s diseases, are currently in progress (Table 2) [88,89,90]. Initial studies in Friedreich’s ataxia patients have shown reduction in excess deposited iron in the brain with reduction in neuropathy and ataxic gait without apparent hematological or neurological side effects following 6 months’ treatment with L1 at 20–30 mg/kg/day [91]. Many clinical studies with L1 involving Friedreich’s ataxia patients are also in progress. Several studies investigating the effect of L1 in NBIA patients, which included a placebo-controlled double-blind multicenter trial in PKAN patients, demonstrated radiological improvement with reduction of iron load in the basal ganglia and a trend to slowing of disease progression [92,93,94,95,96]. Similarly, in two recent independent clinical trials in Parkinson’s disease patients, L1 has been reported to slow disease progression and also to improve motor function [97,98].

The antioxidant potential of L1 has also been investigated in other non-iron loaded categories of patients, where other organs are affected. In preliminary studies using L1 (50 mg/kg/day) in 53 diabetic and non-diabetic glomerular disease patients for 6–9 months have shown a marked, persistent drop in the mean albumin/creatinine ratio and stable renal function in diabetic patients and non-significant reduction in urinary protein with no significant changes in serum creatinine in non-diabetic patients [99].

### 3.2. Targeting of Oxidative Stress Toxicity, Ferroptosis and Increase in Serum Ferritin Production by Deferiprone

Under normal conditions, oxidative stress toxicity can be controlled by the antioxidant defense system present in the organism and also the consumption of dietary antioxidants. However, this balance is not observed in many clinical conditions of free radical pathology including those involving excess toxic iron, where the production of reactive oxygen species and oxidative stress toxicity is on the increase. The increased production of reactive oxygen species by toxic iron forms can potentially damage almost all organic biomolecules including lipids, nucleic acids, sugars and proteins, leading progressively to subcellular, cellular and tissue damage in many diseases [58,100,101]. In each of these diseases, the pathological changes including tissue damage can be reversible or irreversible but can also lead to other effects such as chronic inflammation and carcinogenesis [58,100,101,102,103].

A major area of drug targeting strategy against COVID-19 involves the fatal and other serious toxic side effects of the disease, including all stages leading to hyper-inflammation, which follows the infection by SARS-CoV-2. Different drug targeting strategies could be designed for the inhibition of one or more of the other stages following hyper-inflammation, including the vicious cycle of oxidative stress toxicity, endothelial damage, acute respiratory distress syndrome (ARDS), hypoxia, multi-organ and systemic failure and sepsis [3,14,15,104].

The antioxidant potential of L1 has been previously established in many and different stages of oxidative stress toxicity using in vitro, in vivo and clinical models [57,58,68,69,74,81,82,91,92,93,94,95,96,97,98,99,100,101]. In this context, L1 is considered as one of the most potent antioxidant drugs in clinical use [58]. Combination of L1 with other potent antioxidant drugs such as N-acetylcysteine, which has a different mechanism of antioxidant activity and also different targets related to oxidative stress toxicity, appears to offer better and more effective antioxidant therapeutic options against COVID-19 [3,5,58]. In particular, endothelial cell protection has been shown by L1 in clinical and non-clinical studies of endothelial damage, which is a prime target for decreasing mortality in COVID-19 [77,78,81,105].

Many other specific metabolic pathways could be targeted by iron chelators involved in COVID-19. In particular, iron chelators could target ferroptosis, which is a newly identified form of cell death directly linked to iron toxicity and established in different cell types, including those affected by COVID-19 [106,107,108,109]. Ferroptosis is a form of oxytosis caused by free radicals arising from iron and involves transcription and other factors. It is mainly characterized by the accumulation of lipid peroxides and an iron-dependent autophagic cell death program, which is different from apoptotic and necrotic cell death. A major characteristic of the autophagic process in ferroptosis is the liberation of increased iron through ferritinophagy, which accelerates cell damage [110,111,112]. Under normal conditions, ferritinophagy involves the sequestration of ferritin into autophagosomes and its delivery to lysosomes for degradation. Different factors influencing the process of autophagy and lysosomal degradation can also affect ferritinophagy and ferroptosis [113,114,115,116]. Ferroptotic cell death is evident in many clinical conditions including cardiac and multi-organ damage in COVID-19 [117,118,119].

Several other pathways of iron metabolism in addition to ferroptosis and oxidative stress toxicity have been shown to be directly affected and contribute to cellular and tissue damage in COVID-19. These changes include increased production of serum ferritin, “hyperferritinemia”, and localized iron overload in cells involving primarily macrophages, mitochondrial damage and decrease in hemoglobin resulting in hypoxemia and also systemic hypoxia [120,121,122,123,124,125].

Ferritin is an iron storage protein, which can store up to 4500 molecules of iron and is found in all cells and also in serum. It increases in iron overloading diseases and decreases in iron deficiency [126,127]. Serum ferritin contains negligible amounts of iron and is used as a diagnostic tool for the body’s iron stores. Ferritin can play other roles in addition to storing iron, including that of a signaling molecule and as a modulator of the immune response [128,129,130,131]. It can also act as an acute phase reactant, as well as a regulator of cytokine synthesis and release, which are responsible for the inflammatory storm observed in COVID-19 [123,124,125,128,129,130].

Hyperferritinemia is a negative prognostic factor for any disease and it can also be a result of other forms of inflammation besides that observed in COVID-19, such as autoimmune disorders, infection and malignancy [126,127,128,129,130,131]. The immune response in all these conditions leads to activation of macrophages, iron uptake into macrophages and reduction of serum iron, and with concurrent increased synthesis and secretion of ferritin in plasma by macrophages [108,109,110,111,112,113]. The uptake of increased amounts of iron by macrophages is accomplished via transferrin receptors, erythrophagocytosis, hemoglobin catabolism, ferritinophagy and damaged cells [132,133]. Reduced normal function including reduced antiviral activity of iron laden macrophages and also progressive tissue damage is observed in COVID-19. In contrast, the increased iron uptake in macrophages and reduction in serum iron results in iron deficiency in other cells, where concomitant reduction of enzymatic activity in many iron metabolic pathways can cause general reduction in normal cell function and activity including apoptosis [133].

The reduction of the antiviral activity of macrophages against COVID-19 due to the increased uptake of iron, as well as the associated iron deficiency in normal cells, may possibly be restored by L1. In particular, L1 is the most effective drug of iron mobilization from ferritin and it has also been shown to remove iron from many cells types including macrophages [134,135,136]. Furthermore, L1 has been shown to remove iron from the reticuloendothelial system in inflammatory diseases such as in anemic rheumatoid arthritis patients and subsequently cause an increase in the hemoglobin levels of the patients [137,138]. The mode of the “buffering” activity of L1 involves the iron release from iron loaded reticuloendothelial cells, exchange of iron with transferrin and increase in transferrin saturation with subsequent donation of iron to the erythropoietic cells for the production of hemoglobin and also other cells with reduced iron stores.

Substantial reduction in serum iron and substantial increase in serum ferritin are observed in seriously ill COVID-19 patients, similar to rheumatoid arthritis patients and other patients with the anemia of inflammation or anemia of chronic disease [111,113,115]. In addition, increased deposited iron is also observed in alveolal macrophages in COVID-19 patients [115]. In this context, it is anticipated that the “buffering” activity of L1 by the removal of iron from iron loaded macrophages and its donation to transferrin, as well as the redistribution of iron involved or released in tissue damage observed in the lungs and other organs of COVID-19 patients, could potentially reduce serious illness and fatalities. Furthermore, inhibition of the release of toxic iron, associated oxidative stress toxicity and ferroptosis may potentially decrease other associated tissue damage pathways involved in COVID-19 [139,140,141]. In this context, iron chelating drugs and especially L1, which can permeate cells, mobilize toxic iron from different cellular compartments and inhibit associated toxicity, as well as inhibit ferroptosis, can be considered as the first line for the treatment of COVID-19 and many other diseases where ferroptosis has been implicated [139,140,141].

### 3.3. Targeting Hypoxia in COVID-19 by Deferiprone

The major symptoms and cause of mortality in COVID-19 patients are mainly associated with a respiratory syndrome and lung damage, respiratory failure and hypoxia. Seriously ill COVID-19 hypoxic patients have lung damage that causes a low level of ratio of peripheral blood oxygen saturation to fraction of inspired oxygen and if they cannot receive early successful treatment, only about half of the patients can survive with the aid of mechanical ventilation [142,143]. Drugs that can cause inhibition or reduction of the acute respiratory distress syndrome and prevent hypoxia can reduce substantially the rate of morbidity and mortality of COVID-19 patients [144].

At the cellular level, hypoxia in COVID-19 patients involves only lowering of oxygen levels without affecting glucose and other nutrient availability. Drugs activating the anti-hypoxic response at the cellular level may improve the hypoxic state of COVID-19 patients. The identification of the hypoxia inducible factor (HIF), a transcription factor which switches the cell from aerobic to anaerobic glycolysis was found to play a key role in hypoxia and to be considered as a possible target for therapeutic drugs against COVID-19 [145]. In particular HIF, is a substrate of HIF prolyl hydroxylase, which among other functions activates a group of genes participating in glucose metabolism. Hydroxylation appears to be the main regulator of HIFα subunit protein stability and is controlled by HIF prolyl hydroxylases (HIF PHD) [146]. One of the major targeting prospects of iron chelators in relation to hypoxia in COVID-19 is their ability to inhibit HIF PHD, which are non-hem iron-containing dioxygenases [147,148,149]. In this context and many other cases of iron-containing enzymes, iron removal or displacement by chelators can reduce the activity of HIF PHD and increase the anti-hypoxic response, thus improving the hypoxic state of COVID-19 patients [150].

In addition to their anti-hypoxic response, HIF PHD chelator inhibitors have the potential for the treatment of anemia by enhancing the production of endogenous erythropoietin and increasing erythropoiesis [137,138,151,152]. Furthermore, hydroxylases are involved in a variety of other functions such as collagen synthesis and the hydroxylation of dopamine to nor-epinephrine, where the regulation of iron and other cofactors such as ascorbic acid are important parameters in the treatment of associated diseases [153,154]. 

Effective inhibition of hydroxylases has been observed using L1 and other chelators in vitro in vivo and clinical studies. In particular, inhibition of the di-iron-containing deoxyhypusine hydroxylase by L1 has been related to antiviral activity.

### 3.4. The Antiviral Effects of Deferiprone

A major advantage of the possible development of L1 against COVID-19 is its antiviral properties, which were identified many years ago in several viral species, but only recently clinically tested against human immunodeficiency virus (HIV). There are different mechanisms by which L1 can exert its antiviral effects, which are mainly related to iron chelation but also to other metal binding, as well as other structural characteristics.

Viruses and other invading microbes require iron for their survival and are in competition with host cells for the acquisition of iron via different mechanisms [155,156]. Iron-containing enzymes are involved in many important biological pathways including transcription, viral mRNA translation, and viral assembly, DNA synthesis, ATP generation, etc., all of which are important processes required by viruses including SARS-CoV-2 to replicate in host cells [3,5,155,156]. Iron overload is a negative prognostic factor for all diseases including viral and microbial infections. In this context, targeting iron removal by chelation and limiting iron availability to viruses and other microbes is a therapeutic strategy for the treatment of many diseases including SARS-CoV-2 infection [3,5,139,157,158,159,160].

One of the antiviral targets identified for L1 is similar to HIF PHD inhibition and is related to the inhibition of a retroviral protein containing hypusine, a lysine-derived hydroxylated residue. It appears from in vitro cell studies that the inhibition by L1 is achieved by binding iron required by the iron-containing enzyme hypusine-forming deoxyhypusyl hydroxylase in T-lymphocytic and promonocytic cell lines, thus suppressing replication of HIV-1 [161,162]. The general mode of antiretroviral action by L1 was suggested to be the selective suppression of retroviral protein biosynthesis via inhibition of HIV-1 gene expression and triggering preferential apoptosis of retrovirally infected cells [163,164]. In this context, the pathway of therapeutic ablation of pathogenic cells markedly improves the outcome of infected HIV patients and also of patients with many other viral diseases.

Several other studies followed confirming the antiviral effects via PHD inhibition by L1 and related alpha-ketohydroxypyridine iron chelators such as ciclopirox and the naturally occurring chelator mimosine, as well as other iron chelators including DF [165,166,167]. Similar antiviral effects of L1 targeting hypusine-forming deoxyhypusyl hydroxylase have been shown in other viruses including human T cell leukemia virus type I (HTLV-1) and mouse mammary tumor virus (MMTV) [168].

Further insights into the mode of antiviral activity of L1, as well as on clinical parameters including the optimal concentration of L1 needed for antiviral activity, duration of action and safety, were obtained from clinical studies of L1 in HIV-1 infected patients [169]. In brief, in HIV isolate-infected cell cultures, L1 caused decline of HIV-1 RNA and HIV-1 DNA and also apoptotic DNA fragmentation. The L1 treatment with estimated threshold at about 150 μM did not allow viral breakthrough for up to 35 days on-drug and at least 87 days off-drug viral rebound, indicating resiliency against viral resistance. In a double-blind, placebo-controlled, randomized exploratory trial in 14 HIV-1 infected asymptomatic patients, who attained the threshold concentrations in serum and completed the protocol of 17 oral doses in a week of L1, there was a decline of HIV-1 RNA on-drug that was maintained off-drug for 8 weeks. Seven patients received 33 mg/kg three times daily and 7 patients, as well as 6 normal volunteers, 50 mg/kg three times daily doses of L1, and 6 including 2 normal volunteers received placebo. With regards to safety and tolerability, 86% of the subjects tolerated the 99 mg/kg daily dose and 61% the 150 mg/kg daily dose, which is one and a half times higher than the FDA-approved daily dose in iron loaded thalassemia patients. Adverse events were of a hematological, gastrointestinal and hepatobiliary nature with primary toxicity being the increase in serum liver enzymes. The clinical studies suggested that L1 was the first low molecular weight drug that offered the prospect of reducing the pool of cells that harbor infection-relevant HIV-1 DNA [169].

The inhibition of several other iron- and also other metal-containing enzymes can be targeted by L1 in relation to SARS-CoV-2. This includes the inhibition of ribonucleotide reductase, an iron-containing enzyme involved in DNA synthesis, which is a major target for cancer therapeutics but also for antiviral activity because it is necessary for viral replication [157]. Similarly, several other metal ion-containing proteins are involved in the replication and transcription of SARS-CoV-2, including RNA-dependent RNA polymerase, which is a key manganese-containing enzyme catalyzing the synthesis of viral RNA and thus plays a central role in the replication and transcription cycle of the virus. Although L1 is a weaker chelator for manganese and zinc in comparison to iron, binding of the former two metals may affect viral replication [170].

Different antiviral mechanisms against SARS-CoV-2 may also be implicated in relation to the chemical, molecular weight and structural characteristics of L1, which has many similarities to the natural pyrimidine nuclear RNA bases of uracil and cytosine [171,172]. It is envisaged that such resemblance may result in inhibition similar to that observed by remdesivir, an antiviral drug with broad spectrum antiviral activity used in COVID-19 patients. Remdesivir is a pro-drug metabolized to a ribonucleotide analogue inhibitor of viral RNA polymerase [3,5,17,18,19].

The multi-targeting antiviral effects of L1 against viral infections may contribute to the overall therapeutic potential for application at different stages of the SARS-CoV-2 infection and also related side effects [173,174]. Further studies and clinical trials are needed to characterize the antiviral effects of L1, including anti-SARS-CoV-2 activity.

### 3.5. The Prospects for the Use of Deferiprone against Microbial Infections and Sepsis in COVID-19

Microbial infections and sepsis are important contributory factors that have caused an increased number of fatalities during the COVID-19 pandemic, with no available effective antimicrobial or other therapy and additional related difficulties including ineffectiveness of antimicrobial drugs which are caused by antibiotic drug resistance [3,158,175]. Sepsis is caused by many microorganisms including bacteria, viruses and fungi and affects initially major organs including the lungs, brain, urinary tract, skin and organs of the abdominal cavity. It usually affects people with different clinical conditions and a weak immune system including COVID-19, cancer and diabetes. Among other changes, sepsis is characterized by deregulation of immune response, iron metabolic changes and increased levels of oxidative stress toxicity [3,176,177,178,179]. 

Iron is essential for the growth and proliferation of all microbes and the progression of sepsis [180]. Under normal conditions, the control of iron by the host is considered a major component of innate host defenses. In many cases this is accomplished by the withholding of iron by transferrin in plasma. However, microbes produce potent iron chelators termed siderophores, which compete with transferrin for the acquisition of iron to support their growth and proliferation [150,181]. 

An outbreak of the fungal disease mucormycosis or “black death” in COVID-19 patients was reported in different countries and in particular India during the pandemic [182,183,184]. Deregulated iron homoeostasis, hyperferritinemia and hyperglycemia in COVID-19 patients appears to favor the growth of different *Zygomyces* species causing mucormycosis [185,186,187]. The acquisition of iron from the host is essential for the growth and development of mucormycosis, which is mostly accomplished from the production of the siderophore rhizoferrin by the fungus [184,185,186,187].

It appears that selected chelating drugs inhibiting effectively the uptake of iron by microbes can play a major role in the targeting of infection and sepsis in COVID-19 and also provide alternative therapies in patients with antibiotic drug resistance [175,181,188,189]. However, in contrast to inhibiting microbial growth, some chelating drugs can have the opposite effect and exacerbate infections, mainly by acting as siderophores for specific microbes. For example, yersiniasis and mucormycosis are among the serious toxic side effects of deferoxamine, which can occur in iron loaded and renal dialysis patients, respectively. In both cases, deferoxamine appears to act as a siderophore and donates iron for the growth of *Yersinia enterocolitica* and *Zygomyces*, respectively [190,191]. 

In contrast to deferoxamine, both L1 and deferasirox do not appear to promote the growth of either *Yersinia enterocolitica* or *Zygomyces* and have been tested for the treatment of mucormycosis with encouraging results [192,193]. Furthermore, the antibacterial activity of deferoxamine, L1 and other chelators, which has been previously tested, appears to be chelator concentration-dependent [193]. More recently, the alpha-ketohydroxypyridine chelating drug ciclopirox, which has been clinically developed for the treatment of external fungal and other infections of the skin and nails, has also shown promising antimicrobial effects [194]. Similarly, the naturally occurring antifungal chelator pyridinethione (omadine) and its salts have also been used for more than 50 years for infections of the skin [195].

The anti-inflammatory and antimicrobial activities of the chelating drugs deferoxamine, L1 and deferasirox have recently been tested in two murine sepsis models, which partly resemble side effects observed in COVID-19 patients [196]. In this context, endotoxemia and polymicrobial abdominal sepsis were utilized to differentiate anti-inflammatory versus antimicrobial activities of the three chelating drugs and an experimental chelator. In both models, leukocyte adhesion reduction was the most effective via L1 out of the three chelating drugs, and deferasirox was the least effective. Similarly, inflammation in the abdominal sepsis model, which was assessed by cytokine measurements, indicated exacerbation by deferasirox and deferoxamine for plasma interleukin (IL)-6 and reductions to near-control levels by L1. It appears from these two models that L1 has additional advantages over the other chelating drugs for possible use in COVID-19 patients [196].

Promising antimalarial effects have also been observed in in vitro and clinical studies using L1 as monotherapy and in combination therapies [197,198].

**Figure 2:**
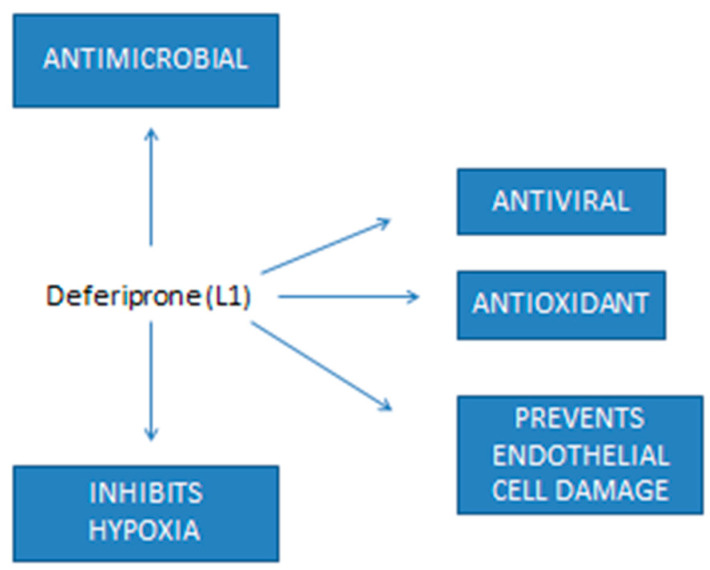
Some of the properties of deferiprone with potential treatment prospects in cases of COVID-19. There are many possible therapeutic benefits of using deferiprone (L1) at different stages of COVID-19, including antiviral activity and also activity against many other serious side effects associated with the high morbidity and mortality of the disease.

There is a lot of evidence to support the clinical application of L1 and other chelating drugs for the emergency treatment of microbial infections and sepsis in COVID-19 and many other fatal diseases. Further studies are required to examine the prospects of clinical application of L1 as monotherapy or in adjuvant therapies against infections and sepsis in COVID-19 patients (Figure 2).

## 4. Conclusions

The COVID-19 pandemic has raised the alarm for the need of humanity to be prepared in the future against viral and other infectious diseases, which can cause high morbidity and mortality rates. New therapeutic strategies should be developed in addition to antiviral drugs and vaccines, which should include many other specialized drugs dealing with the symptomatology of such diseases in order to reduce fatalities. In particular, the need for emergency therapeutics, which include repurposed approved drugs and nutraceuticals, could increase the prospects and facilitate the process needed for emergency treatments in COVID-19 and related diseases.

Iron chelating drugs and in particular L1 have been identified to fulfill many of the requirements of a multi-target therapeutic against COVID-19 and diseases with similar symptomatology (Figure 1). In particular, the antiviral properties of L1 in different viral species and encouraging clinical results in HIV patients support its further evaluation in other viral diseases including its clinical effects against SARS-CoV-2 growth and proliferation. Similarly, L1 has been shown to be effective against many of the side effects of COVID-19 including hypoxia, endothelial cell damage, microbial infections and sepsis. Studies do not support an increase in microbial growth or infection in patients treated with parenteral or enteral iron, and the other potential benefits of iron chelation in the treatment of COVID-19 and other microbial diseases are also encouraging. Furthermore, L1 has been shown to be one of the most potent antioxidant drugs in many in vitro, in vivo and clinical models, supporting its introduction for treatment in many categories of patients with no available effective treatments, such as neurodegenerative diseases and cancer.

The overall clinical effects and safety of L1 in many diseases support its emergency testing and use in COVID-19 patients and other categories of patients with similar symptomatology.

## Figures and Tables

**Table 1:** Properties and mode of action of the chelating drug deferiprone.

Chemical and physicochemical properties
Molecular weight: 139. Molecular weight of iron complex: 470.
Charge of L1 and iron complex at pH 7.4: neutral.
Partition coefficient (n-octanol/water): 0.19 (hydrophilic).
Stability constant (Log β) of deferiprone iron complex: 35.
**Clinical and biological effects**
Recommended dose in different categories of patients including combination with other chelating drugs: 10–100 mg/kg/day.
Effect of deferiprone on iron absorption: decrease of iron absorption.
Iron removal from diferric transferrin in iron loaded patients: removal of about 40% of iron at deferiprone concentrations of greater than 0.1 mM. Iron removal from ferritin and hemosiderin.
Differential iron removal from various organs of iron loaded patients: preferential iron removal of excess iron from the heart but also from liver, spleen and pancreas of iron loaded patients. Efficacy in iron removal is related to dose.
Iron redistribution in diseases of iron metabolism: Deferiprone can cause iron redistribution from iron deposits and also through transferrin from the reticuloendothelial system to the erythron in the anemia of chronic disease. Similar effects of excess iron redistribution is observed in patients with neurodegenerative diseases
Increase excretion of metals other than iron, e.g., zinc (Zn) and aluminum (Al): increased Zn excretion in iron loaded patients, following long-term treatments. Increase Al excretion in renal dialysis patients.
Iron mobilization and excretion of chelator metabolite iron complexes: no iron binding and no increase in iron excretion by the deferiprone glucuronide metabolite.
Combination chelation therapy: Combination therapies of all chelating drugs are more effective in iron excretion than monotherapies. The International Committee On Chelation of deferiprone and deferoxamine combination protocol causes normalization of the iron stores in thalassemia patients.
**Metabolism and pharmacokinetics**
Metabolite(s): The deferiprone−gluguronite conjugate is cleared through the urine but have no iron chelation properties.
T1/2 absorption of deferiprone: 0.7–32 min. T max of deferiprone: mostly within 1 h on empty stomach.
T1/2 elimination of deferiprone: 47–134 min at 35–71 mg/kg dose.
T1/2 elimination of the deferiprone iron complex: estimated within 47–134 min.
T max of the L1 iron complex: estimated within 1 h. T max of the metabolite deferiprone-glucuronide: 1–3 h.
Route of elimination of deferiprone and its iron complex: urine.

**Table 2:** Categories of patients treated with deferiprone (L1).

Categories of patients with iron overload
Beta-thalassemia major
Beta-thalassemia intermedia
HbE β-thalassemia
HbS β-thalassemia
Sickle cell anemia
Myelodysplastic syndrome
Aplastic anemia
Fanconi’s anemia
Blackfan-Diamond anemia
Pyruvate kinase deficiency
Idiopathic hemochromatosis
Iron overload in hemodialysis
Juvenile hemochromatosis
**Categories of patients with normal iron stores**
Renal dialysis
Rheumatoid arthritis
Malaria
HIV
Breast cancer
Prostate cancer
Parkinson’s disease
Alzheimer’s disease
Friedreich’s Ataxia
Neurodegeneration with brain iron accumulation
Pantothenate kinase 2-associated neurodegeneration (PKAN)
Glomerulonephritis and diabetic nephropathy

## Data Availability

Not applicable.

## Abbreviations

COVID-19: coronavirus disease 2019; BPAN: beta-propeller protein-associated neurodegeneration; G-CSF: granulocyte-colony stimulating factor; HIF: hypoxia inducible factor; HIV: human immunodeficiency virus; HTLV-1: human T cell leukemia virus type I; MMTV: mouse mammary tumor virus; MPAN: mitochondrial membrane protein-associated neurodegeneration; L1: deferiprone; MRI: magnetic resonance Imaging; NTBI: non-transferrin bound iron; PKAN: pantothenate kinase-associated neurodegeneration; SARS-CoV-2: severe acute respiratory syndrome coronavirus 2; T1/2: half-life; WHO: World Health Organization.
